# Respite Service Use among Dementia and Nondementia Caregivers: Findings from the Caregiving in the U.S. 2015 Survey

**DOI:** 10.1093/geroni/igab046.3428

**Published:** 2021-12-17

**Authors:** Yura Lee, Wonchan Choi, Min Sook Park

**Affiliations:** 1 Helen Bader School of Social Welfare, Milwaukee, Wisconsin, United States; 2 University of Wisconsin-Milwaukee, University of Wisconsin-Milwaukee/Milwaukee, Wisconsin, United States

## Abstract

Caring for persons with dementia places a significant physical, emotional, and financial burden on caregivers. Although dementia caregivers may be exposed to more challenging caregiving environment than caregivers of those with other chronic conditions, little is known about how specific factors related to respite service use differ between dementia and nondementia caregivers. Thus, this study first examined factors related to respite service use among caregivers and further tested the moderating effect of dementia caregiver status in these relationships using nationally representative U.S. data. Logistic regression analyses were conducted among 1,203 caregivers (276 dementia and 927 nondementia caregivers) from the national Caregiving in the U.S. 2015 data. Caregivers’ race and ethnicity as a predisposing factor, caregivers’ self-rated health as an enabling factor, and care recipients’ living arrangement and functional limitations as need factors were significantly related to respite service use among caregivers. Moreover, dementia caregiver status moderated the association between enabling factors (i.e., household income, work status, and self-rated health) and respite service use. Our findings imply that dementia caregivers may be more in need of respite service use than nondementia caregivers when they have limited enabling factors (e.g., lower household income, nonworking status, poorer health). Policy and practice efforts that specifically support enabling factors are suggested to promote more respite service use among dementia caregivers.

